# Deep Interpolation of Remote Sensing Land Surface Temperature Data with Partial Convolutions

**DOI:** 10.3390/s24051604

**Published:** 2024-02-29

**Authors:** Florian Huber, Stefan Schulz, Volker Steinhage

**Affiliations:** Department of Computer Science IV, University of Bonn, 53121 Bonn, Germany; s6sfschu@uni-bonn.de

**Keywords:** deep interpolation, partial convolutions, land surface temperature (LST), image inpainting, gapless MODIS

## Abstract

Land Surface Temperature (LST) is an important resource for a variety of tasks. The data are mostly free of charge and combine high spatial and temporal resolution with reliable data collection over a historical timeframe. When remote sensing is used to provide LST data, such as the MODA11 product using information from the MODIS sensors attached to NASA satellites, data acquisition can be hindered by clouds or cloud shadows, occluding the sensors’ view on different areas of the world. This makes it difficult to take full advantage of the high resolution of the data. A common solution to interpolating LST data is statistical interpolation methods, such as fitting polynomials or thin plate spine interpolation. These methods have difficulties in incorporating additional knowledge about the research area and learning local dependencies that can help with the interpolation process. We propose a novel approach to interpolating remote sensing LST data in a fixed research area considering local ground-site air temperature measurements. The two-step approach consists of learning the LST from air temperature measurements, where the ground-site weather stations are located, and interpolating the remaining missing values with partial convolutions within a U-Net deep learning architecture. Our approach improves the interpolation of LST for our research area by 44% in terms of RMSE, when compared to state-of-the-art statistical methods. Due to the use of air temperature, we can provide coverage of 100%, even when no valid LST measurements were available. The resulting gapless coverage of high resolution LST data will help unlock the full potential of remote sensing LST data.

## 1. Introduction

Land Surface Temperature (LST) recovered from remote sensing data is an important resource for a variety of tasks, such as yield prediction [1], monitoring the changing climate [2], or analyzing the environmental impacts of different types of land cover [3], just to name a few. LST data are crucial in various different fields of research, as they are freely available nearly worldwide, while providing historical records and high spatial and temporal resolution. A problem with regard to LST records derived from remote sensing data sources is missing observations due to clouds or cloud shadows that block the satellites’ view on the surface and impede a gapless coverage of LST data [4]. One of the leading data sources for LST is the MODA11 product estimated from measurements of the Moderate Resolution Imaging Spectroradiometer (MODIS) provided by NASA to give multiple estimations of LST during the day, also suffering from incomplete records. A different source for temperature estimations is ground-site measurements in the form of local ground-site weather stations that reliably provide records of different climatic attributes for a fixed location. Although these cannot provide the spatial resolution of remote sensing data, because of their fixed location, the reliability of these measurements is nearly flawless. Naturally, the question arises of whether the gaps in the LST records can be filled, and so filling in gaps in remote sensing data LST records is a thoroughly investigated research field. Research regarding the topic has been present since remote sensing data became widely available in the early 2000s, and is dominated by statistical approaches that mostly perform some form of fitting a function to the neighboring valid temperature measurements to estimate missing values in between. For this, the neighboring measurements could be spatially close or temporary close to the missing values. This methodology foregoes the rapid development of deep learning, where the task of image inpainting can be seen as related to the problem of missing LST data. The usual setting for both problems is quite similar, with both having input data consisting of parts with valid information and parts with missing information that needs to be inferred based on what the valid information dictates.

We present a novel deep interpolation approach to provide high-resolution LST data without gaps by combining the spatial resolution of LST derived from remote sensing data with the reliability of local ground-site measurements. In the first step, we convert the air temperature captured by the local weather stations to surface temperature by learning individual linear regression models, aligning the air temperature information with the LST information from the remote sensing data for every ground site. We utilize those linear models to provide a minimum amount of information for each day of the LST data so that every day has a minimum amount of valid data, regardless of cloud coverage. For better readability, we will refer to the LST data covering our research area for a day as an LST image throughout this paper, with each pixel being an individual LST record. Finally, in the last step, we use deep learning in the form of a U-Net consisting of partial convolutions to completely fill in the holes in every LST image. The training of our U-Net is performed by only using partially complete ground-truth LST images. Each image has further pixel information removed by creating a mask from the occluded pixels of a random other image of our dataset. The loss during training is then calculated on every pixel that has LST information in the ground-truth image. The same technique is used to obtain data for testing and validating our approach, where all available LST images without missing information are used and masked with a random existing pattern of non-available information from the rest of the data. This allows us to provide a reliable and comprehensive analysis of the capabilities of our approach.

For our work, we focus on a wine growing region in Rhineland-Palatinate, Germany since the final gap-free dataset is used within the federal KI-iREPro project to predict grapevine yields. By focusing our model on a predefined region of interest, it is able to learn the spatial context of the area and implicitly includes information like elevation, which has influence on the climatic conditions.

Summarized, the contributions of this study are the following:

- First usage of deep learning and partial convolutions for LST reconstruction by merging local weather station and LST data derived from remote sensing.
- Extensive evaluation of our approach reveals that our deep interpolation approach outperforms the state-of-the-art algorithms to fill in gaps in remote sensing LST data in terms of completeness and accuracy for our study area.

### 1.1. Related Work

The interpolation of remote sensing LST images is a widely investigated research area, often relying on statistical approaches to interpolate between close neighbors of missing values. This will be covered in the first part of this section. The second part of the related work will describe the state of general image inpainting with deep learning and why this can be related to providing gap-free estimations of LST.

#### 1.1.1. Estimating Missing Land Surface Temperature

The common idea in most efforts to fill in gaps in LST data retrieved from remote sensing observations is to fit some kind of function to valid pixels neighboring the missing values, either in the spatial or temporal domain. An early adoption of this idea can be found in the work of Neteler [5], which fills gaps in MODIS LST in mountainous environments. The study region is the Central Eastern alps, and gap filling is performed through statistical operations that rely on the valid nearest neighbors of missing data points. Finally, an evaluation against meteorological data measurements reveals R2 correlation values between 0.7 and 0.98. The author concludes that the new reconstructed LST time series is reducing the gap between high-spatial and high-temporal resolution. Metz et al. [6] introduce an approach that reconstructs values in time through local weighted regression with a polynomial of order two and considering the five closest neighbors in time. Afterward, spatial imputation is performed with thin plate spline interpolation, including information on emissivity and elevation. All this is tested on MODIS data to achieve a gap-free coverage of Central Europe, achieving remarkable results in dealing with extreme events, such as a heatwave effecting Europe in 2003. Similarly, Zhang et al. [7] infer the temperature of the surface of the land using a two-step algorithm. The first step consists of data filtering and cleaning, where the missing value for gaps and low-quality pixels is computed utilizing the information from other available data from the same day, i.e., observations from other satellite overpasses. In the second step, the overall yearly temporal trend is used as a basis to calculate the missing residuals by fitting a smooth spline function to accessible valid pixels. They report an average Root Mean Squared Error (RMSE) of 1.88 °C, validated by filling artificial gaps of varying sizes. They highlight that, with their approach, there are no obvious block effects caused by large areas of missing values, which might exist in other LST datasets.

Another popular approach is to transfer the LST data to a different domain to fit an interpolation function. Pham et al. [8] fill gaps in MODIS LST data captured in Australia. They use multidimensional robust smooth regression [9], minimizing the squared error after performing a three-dimensional discrete cosine transform with a manually adjusted smoothing parameter. Validation is carried out against ground-based LST data on real-world gaps, revealing RMSE values between 2 °C and 3.9 °C. Similarly, Liu et al. [10] use the same discrete cosine transform and penalization of least squares to fill gaps in the MODIS LST data. Synthetic gaps are produced by the random blinking algorithm to create evaluation data. An average RMSE of 0.91 °C is reported. Both works have the advantage of not relying on alternative geospatial datasets to fill the gaps and therefore avoid additional uncertainty. Furthermore, both works use a parameterization of the smoothing parameters in a way that retains the high-frequency components and therefore the global spatial patterns of the original LST data.

A three-step hybrid method is proposed by Li et al. [11]. They first use other satellite overpasses from the same day to fill gaps, if possible. This is followed by spatio-temporal gap filling and lastly temporal interpolation using neighboring days. The evaluation was carried out in urban areas within the United States with an RMSE between 2.7 °C and 3.3 °C using artificial masks of missing values taken from other real-world examples for the evaluation. Again, the approach does not rely on other geospatial datasets for gap filling. An included daily merging step that uses other satellite overpasses of the same day further decreases the computational complexity of the problem. Focusing on the reconstruction of eight-day MODIS LST data, Xu and Shen [12] use the harmonic analysis of time series to reconstruct missing values. The validation was carried out by removing the information of 76 pixels and considering them as ground-truth data to calculate a mean absolute error of 1.51 °C. The authors highlight the capabilities of the approach to deal with large and persistent gaps in LST data, while struggling with sudden and extreme changes in the temperature.

The first adoption of machine learning for the reconstruction of missing LST values can also be found in the literature. Li et al. [13] produce a gap-free LST product by combining MODIS and ground-site measurements using random forests, achieving an RMSE of 2.756 °C when validated with data at the ground sites. Insights of their work include a clear improvement of the results for clear-sky conditions when compared with cloudy sky conditions and good performance results, even without the usage of spatial information for the reconstruction. Xiao et al. [14] reconstruct LST MODIS data for Zhejang Province. Missing pixel values are calculated individually with different machine learning models, where eXtreme Gradient Boosting (XGBoost) outperformed the other approaches with an average R2 of 0.95. The authors conclude that the XGBoost model demonstrated stronger learning capabilities and was able to fit the complex relations of the data, in comparison to statistical regression approaches or random forests. Both machine learning-focused works show the capabilities of tree-based approaches but have the drawback of needing to interpolate every missing value individually.

Overall, we see that the landscape of filling gaps in LST data focuses on statistical approaches and slowly starts to adapt machine learning methods into the process. A common drawback for all related work is the need to fill every missing value individually, either by fitting some kind of interpolating function or evaluating some kind of machine learning algorithm. This can be improved by our idea of using deep learning to solve the problem. Regarding the type of evaluation, the different approaches vary widely, making a direct comparison difficult. We analyzed the work of Metz et al. [6] to be an excellent execution of the statistical approach to interpolating LST data, and we will use this to obtain a baseline score for our use case and provide a fair evaluation.

#### 1.1.2. Deep Learning for Image Inpainting

Our idea of using deep learning to fill in gaps in MODIS LST data is highly influenced by general image-painting problems. The inpainting of images, in general, refers to a problem in which information about the input image is missing in some way or another. The task is then to infer the missing information in a way that is most likely, given the existing valid pixel information. It has been widely used in different applications to reconstruct image data. Many approaches focus on inpainting regular shapes such as boxes in the center of an image [15,16]. This inherently causes the network to be induced with bias that is usually not wanted in practice, and also something that, in general, rarely happens in the real world. PatchMatch by Barnes et al. [17] has long been the state-of-the-art method for image inpainting. PatchMatch suggests a random sampling base algorithm that computes patches that look similar to the holes to be filled in an image. However, the proposed algorithm only works well as long as there is a patch that is similar to the one already existing, as otherwise, the results will quickly turn out to be visually unappealing and unnatural. Liu et al. [18] introduced partial convolutions in 2018 to overcome the challenge of filling irregular holes. The method does not need to compute similarity metrics for patch-based inpainting but rather just masks out invalid pixels during the convolutional step so that, rather than using wrong data, no data are used. After each convolution, the number of unfilled pixels decreases. The results have shown a huge improvement in the visual appearance, and it was one of the first papers to obtain good results for irregular holes. Furthermore, Han and Howe [19] use 3D partial convolutions in the context of 3D histograms. Histograms depict taxi pick-ups and bike-sharing data in New York. They use a custom 3D variant of the U-Net introduced by Ronneberger et al. [20]. One of the few studies using image inpainting for geoscience tasks is conducted by Sun et al. [21]. They implement a coarse-to-fine task-driven neural network that preserves good visual appearances but is also more suited to geoscience tasks where visual appearance is not as important as predicting specific values. Hence, a coarse model predicts the general appearance and a refinement net refines these predictions.

None of the previous works consider LST data as input for an image inpainting network. This leads to unsolved challenges, such as images consisting of only missing values that need to be interpolated. We solve this problem by the inclusion of ground station data within our work and give a proof that the patterns learned by deep learning architectures can help to produce precise inference of missing LST data by learning the inherent climate patterns of the interest region.

## 2. Materials

The data used in our study are taken from two 2 main sources. On the one hand, we have LST data within the MODA11 product with gaps that need to be filled, and on the other hand, we have local ground-site weather stations that record air temperature within the area. In the following section, we first describe our study area followed by in-depth descriptions of the MODA11 product and the local ground-site weather stations.

### 2.1. Study Area

**Figure 1:**
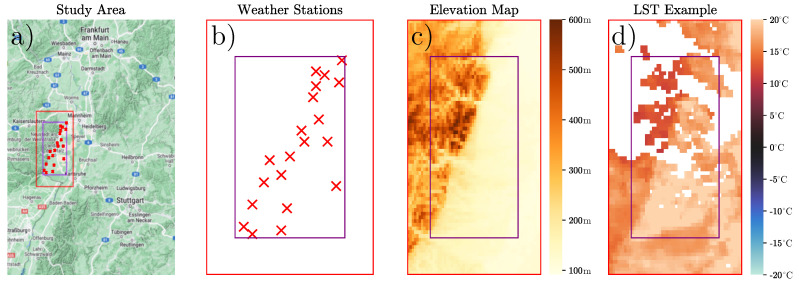
Overview of the study area, weather stations, elevation, and occluded LST data. (**a**) The study area is located mostly in Rhineland-Palatinate, Germany, measuring 47.49 km by 36.96 km with the extended padding shown as a red rectangle and the weather stations shown as red crosses. (**b**) The relative location of the weather stations. (**c**) Elevation map showing a gradient from the lower left corner to the upper right corner. (**d**) An example of LST data on 2 September 2010 showing gaps in the data and the characteristic temperature distribution dictated by the elevation.

The study area is motivated by an ongoing project supported by the Federal Ministry of Agriculture (BMEL) to predict grapevine yields in Rhineland-Palatinate, Germany. The area measures 47.49 km by 36.96 km and is centered around 8.112° N and 49.25° W. It includes vineyards that should be monitored throughout the year. An overview of the location in the south-west of Germany can be seen in Figure 1a, where we see that it is located south of Frankfurt am Main and north-west of Stuttgart. To improve the interpolation of missing values inside the study area, we added a 10 km padding around the relevant area, which will not be considered when evaluating the accuracy of the different interpolation methods. In Figure 1, the original research area is represented by a purple rectangle, while the red rectangle shows the extended area that includes the padding. The study time frame is set from 11 March 2008 to 15 November 2022, with the start of the study time frame dictated by the availability of ground-site weather station air temperature data and the end of the time frame determined due to the availability of remote sensing data at the time of our study. The relative location of the weather stations is shown in Figure 1b. The region itself offers some interesting topology, with heights ranging from 91 to 599 m above zero, including a steep change in elevation along an axis from the south-west to the north-east corner as shown in Figure 1c.

### 2.2. MODIS Data and Occlusions

Our source for remote sensing LST images is the well-known MOD11 product [22]. The LST data are retrieved from a multitude of inputs from the MODIS sensor attached to the Terra satellite provided by NASA by using the generalized split window algorithm [23]. In summary, the algorithm consists of three steps. First, cloudy pixels are detected and skipped in LST production. Second is the estimation of atmospheric column water vapor and lower boundary temperature from seasonal climatological data to improve the accuracy of LST. Third, the estimation of band emissivities, correcting for atmospheric effects at specific wavelengths, is used to finally retrieve the LST information. Other existing data sources for LST records include the Landsat collection that provides LST records at a high spatial resolution but lacks the daily coverage of MODIS that is important for a variety of tasks.

In our case, the data are downloaded using the Google Earth engine [24]. The images were taken with a spatial resolution of 1 km. An example of a partial LST image recovered from the MODIS sensor is shown in Figure 1c. To have as much usable data as possible, we did not consider the quality indicator to remove any further values, as early experiments indicate that, in general, every pixel with a value attached can be considered good enough to be used for further processing. Together with the LST, the product provides information on the emissivity of each pixel. The emissivity is considered when evaluating statistical approaches for gap filling but do not show any added benefit for our approach. The satellite regularly observes our study region around 11:00 a.m., monitoring temperatures from −32 °C to 46 °C with a mean temperature of 12.69 °C, a median temperature of 12.59 °C and a standard deviation of 10.3 °C when considering all valid pixel values in our research area. When considering all 5706 observations in our time frame, 33.24% of all pixels are filled with valid information. This includes a total of 280 images where all 4012 pixels are valid and 1500 images where no pixels are valid. On average, each image consists of about 1507 valid pixels that can be used to fill the values of the remaining 2505 missing pixels.

### 2.3. Ground-Site Air Temperature

To be able to fill the gaps in the LST data, even when the entire padded study area is occluded, we decided to include air temperature measured by local ground-site weather stations in our pipeline. Air temperature and LST are of course different measurements with varying records up to multiple °C, but often show a high correlation [25]. A local ground-site weather station cannot monitor the varying climate in an extended region but excels at providing precise and reliable information about a specific location. Using both weather stations and remote sensing data allows us to combine the best characteristics of both data sources, with the aim of providing reliable LST information with high resolution. We use 20 weather stations in our study area with their locations shown in Figure 1b, located mainly in the low-lying regions of our research area. Weather stations can capture a multitude of features at an hourly rate. We are mostly interested in the data captured at the time when the MODIS satellite observes the region, together with the data from a few hours earlier, because both can give information on how the measured air temperature can be homogenized with the surface temperature that is of interest to our study. We use the average, minimum, and maximum air temperature, together with the air humidity captured at 6:00 a.m. and 11:00 a.m. to infer the surface temperature at 11:00 a.m. of the pertaining day. All features are measured twice, once at a height of 20 cm and once at a height of 200 cm. A list of all weather stations and their coordinates is given in Table 1. Data are captured on account of the federal state Rhineland-Palatinate, Germany [26].

## 3. Methods

In this section, we describe the three main ideas of our approach. First, we describe how we use individual-trained linear regressions to obtain surface temperature approximations for missing LST data when a ground-site weather station is available. Second, we describe the U-Net structure and partial convolutions that perform the interpolation of all other missing values, and third, we describe how we use a partially computed loss function for neural network training to obtain a capable model without the use of non-occluded LST representations with no missing data. We also note that throughout the whole process, we exclude each 140 LST images with full coverage for validation and testing, so they are not used during creating the linear regression models or the training of the network.

### 3.1. Linear Regression for Homogenization of Air Temperature

We train a new linear regression model for every individual ground-site weather station. It is not possible to integrate the data captured from the stations directly into the LST images, as the measured air temperature usually deviates from the surface temperature, although the two are closely correlated. To obtain data to train the linear regression models, we first search for all valid LST information regarding pixels that cover the same area as the respective ground-site weather station. This leaves us with about 1000 to 1600 entries depending on the weather station. An outlier is the Freimersheim station with only 438 valid entries in the training data because it is available only from 1 January 2019. For each station, we choose to use 80% of the data for training and 20% of the data to validate and adjust the process. Note again that the final testing data are excluded throughout the process, and the interpolated LST values are part of calculating the final accuracies of all the approaches tested. A total of eight features are used as input to estimate the LST as described in Section 2.3. The RMSE for homogenizing the air temperature with LST is around 2.5 °C for each station, with the correlation coefficient R2 around 0.9. An explanation of both evaluation metrics can be found in Section 4.3 and an exact listing of the results is shown in Table 1. Experiments with more advanced machine learning models like random forests or eXtreme Gradient Boosting (XGBoost) have not shown any improvements, resulting in us choosing linear regression due to its fast training and evaluation together with high robustness. The good results of the linear regression can be explained by the high correlation between the surface and air temperature, allowing the simple linear regression to obtain the same results as the more complex models.

### 3.2. Partial Convolutions for Interpolating Temperature

**Figure 2:**
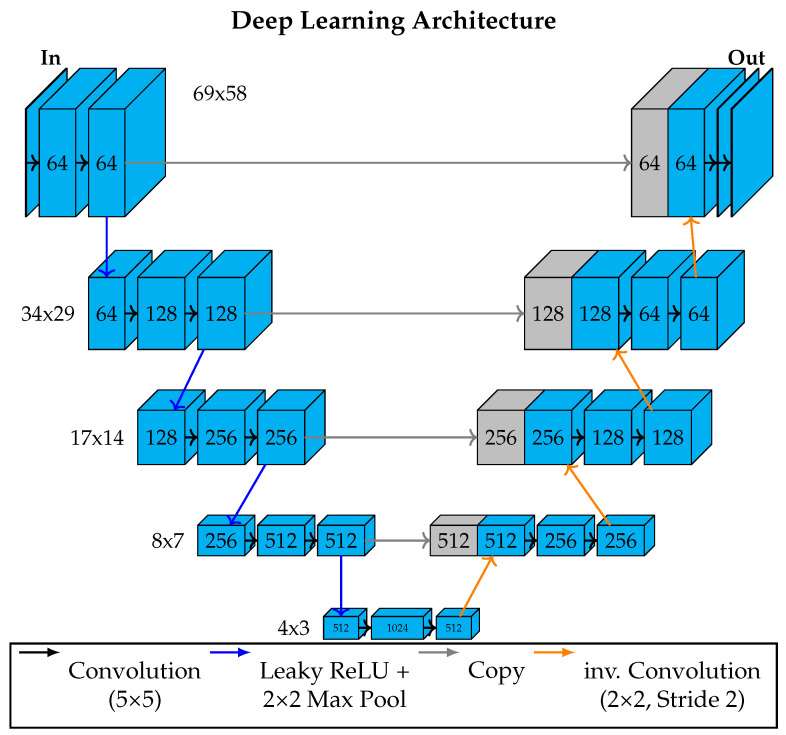
Visualization of the U-Net structure, including partial convolutions used to interpolate gaps in the LST data. This deep learning architecture allows the use of the power of convolutions, while simultaneously obtaining outputs of the same dimension as the input. Furthermore, each step of the downward path allows the pixel values to be estimated according to the surrounding valid pixels.

We propose a U-Net-based architecture inspired by the work of [20] to fill in the gaps in the LST data. The U-Net architecture combines the strengths of convolutions for feature extraction together with the possibility of obtaining an output image of the same dimensions as the input. Furthermore, we can make use of the overlapping tile strategy implemented with the U-Net as explained by [20]. In the original work, this means that the segmentation of an area (tile) is improved when the network has access to the surrounding information (overlap). For our case, this means that pixels that are spatially close to missing values can be used for the interpolation process. The general structure of the network is shown in Figure 2. The input and output of the image consist of an LST image measuring 69 × 58 pixels. At each level of the U-Net architecture, we perform two concatenated 5 × 5 partial convolutions to extract features, shown by black arrows in Figure 2. Partial convolutions as introduced by [18] work the same as regular convolutions, except that they do not include masked pixels. Given convolution filter weights *W*, the input features for the current convolution window are written as *X*. *M* is the binary mask that indicates missing values in *X*. The bias is *b*, and the constant maximum number of possible valid pixels in one convolution step is written as *c*. For each $x^{\prime }$ of the output, a partial convolution can be described as:

(1) $$x^{\prime }=\left\{\begin{array}{ll} W^{T}\left(X⊙M\right)\frac{c}{sum(M)}+b, & \mathrm{if}\;sum(M)>0,\\ 0, & otherwise. \end{array}\right.$$

Here, ⊙ is the elementwise multiplication. The fraction in this equation can be interpreted as a scaling factor for the amount of valid pixels in the calculation. The condition sum(M)>0 checks if at least one valid pixel is available. The mask is updated under the same conditions. If sum(M)>0, then $M^{\prime }$, the new mask, obtains a valid pixel at that position. After the first set of convolutions, the downward path starts with a combination of a leaky Rectified Linear Unit (ReLU) [27] and a 2 × 2 max pooling layer [28]. Leaky ReLU is an activation function similar to a standard ReLU activation. With a standard ReLU activation, all negative values are suppressed, and positive values are propagated linearly with a slope of 1. A leaky ReLU treats positive values the same, but negative values are not suppressed but linearly discounted. The max-pooling step selects the maximum value from a 2 × 2 window. On the one hand, this helps to select the most important extracted features in each step, and on the other hand, this helps us to interpolate missing values, as one valid value can be selected to represent up to three missing values. The application of a leaky ReLU and the max pooling is shown by blue arrows in Figure 2. The lowest layer of our U-Net is called the bottleneck and consists of image tensors of the size 4 × 3. The small size guarantees that after an empty LST image is filled with up to 20 valid pixel values extracted from ground-site weather stations, there are no more missing values in the bottleneck and therefore, there are no missing values throughout the whole upward path and, subsequently, no missing values in the final output image. The stepwise increase in image size in the upward path is carried out by so-called inverse convolutions [29] with a 2 × 2 kernel and a stride of 2, shown as orange arrows in Figure 2. Lastly, at every step of the upward path, we copy the state of the last output of the same layer of the downward path and concatenate the input to the first convolution of the upward path. This is also standard practice for designing U-Net structures and helps stabilize the training process. Finally, the network output is smoothed with a Gaussian kernel of size 5 with a standard deviation of 0.7. This further improves the results, as LST images are mostly rather smooth, while the network output can have steep differences between neighboring pixels.

### 3.3. Learning on Occluded Data

**Figure 3:**
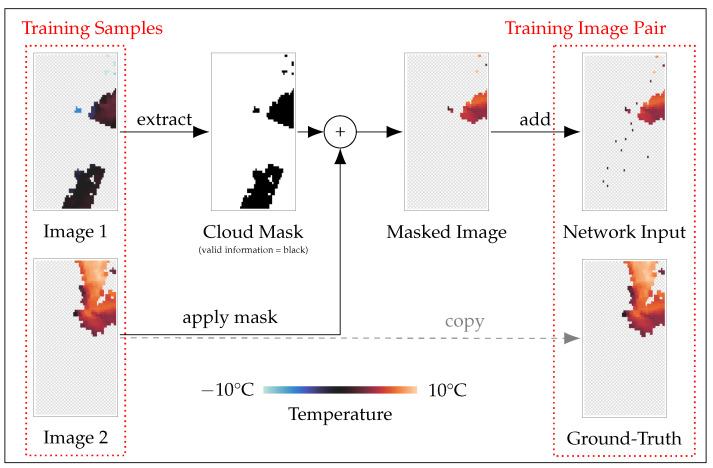
Overview of creating image pairs for network training. Two random training images are selected, one for extracting a real-world example for missing values and one for ground-truth temperature data. The second image is masked according to the missing values in image 1. The network input is then completed by adding LST data, where weather stations are available.

Machine learning and especially deep learning approaches are data driven and need access to pairs of data to learn the underlying patterns of the task at hand. A data pair for training consists of the input and the desired ground-truth output of the network. The input should be the same as the input data for later prediction tasks when the model is fully trained and the ground-truth output represents the desired output for the perfect model. For the estimation of LST data, this means that the input should be an LST image of our region with cloud occlusions, and the ground-truth output would ideally be a non-occluded version of the same image, having access to the LST information, even for the occluded pixels of the input. The network can then learn gradually how the occluded pixels should be interpolated, given the remaining input by trying to match the ground-truth output as close as possible. However, fully available ground-truth data for LST reconstruction are very limited, and we need a different solution to train our network. We solve the problem using partially occluded ground-truth data. This means that even our ground-truth images showing the desired output of the network have missing pixel information. The network, however, will output a fully reconstructed LST image for every input. This discrepancy can be addressed by implementing only a partial loss function that will evaluate the network output, based on whatever pixels are available in the ground truth. The idea is that with a significant database of training instances, every pixel is present within the ground truth for a portion of the training, and therefore the network will learn how to estimate the value of every pixel in our region over time. To create pairs of input and output data that can be used to train the network, we took all partial LST images available for our study and removed additional pixel information from each image. To create training images that resemble the real-world conditions the best, we take the pattern of occluded pixels (cloud mask) from a random different image of our training dataset. This often results in some pixels being masked, where ground-truth data are available, which can ultimately be used to estimate how well the network can fill gaps in LST data. An overview of the process is shown in Figure 3. Summarized, the process of creating a pair of data for network training consists of four steps. First, we sample two random images from our database, both of which have partially occluded information. Second, we extract the cloud mask as a possible pattern of real-world occlusion from one of the images. Third, the cloud mask is applied to the second selected image to mask further pixels as invalid. And lastly, the pixels representing the ground-site weather stations are filled with the relevant information and the resulting processed image is the network input, while the original second image becomes the ground-truth data for network training. The average amount of non-valid pixels in the training images is increased throughout this procedure from 2505 non-valid pixels to 2729 non-valid pixels. The increase in those numbers is to be expected, as the amount of non-valid pixels per image can only be increased for every individual image. Throughout this process, we also create training images, where no additional pixels are made invalid, resulting in the output image not having any additional information compared to the input images. However, including those images in training the network is beneficial, as the network can use those to learn the spatial dependencies of the LST images in our study region and therefore further improve accuracy and stabilize training. During the training process, we use the Mean Squared Error (MSE) loss between the ground truth *y* and the prediction $\hat{y}$: $L\left(y,\hat{y}\right)=||y-\hat{y}\left|\right|$. To overcome the problem of missing ground-truth data for an entire image, the MSE is calculated only where the ground truth has viable information. We note that this includes both pixels that exist in the network input and pixels that are occluded in the network input image, providing stability during the training process. For the final evaluation of the test data, only pixels newly inferred by the network are used to calculate the error metrics.

## 4. Experimental Results

In this section, we first describe a state-of-the-art statistical approach to fill gaps in LST data, followed by a comparative evaluation to show the capabilities of our approach.

### 4.1. A Statistical Approach to Interpolating Remote Sensing Data

As a competitor and to evaluate the capabilities of our approach, we opted to use our own implementation of a state-of-the-art procedure for the statistical interpolation of LST introduced by Metz et al. [6]. They present their approach to fully reconstructing LST data for Central Europe at a resolution of 1 km in a two-step process, even including elevation and emissivity. The elevation helps to recognize temperature patterns induced at different height altitudes and can be used, regardless of how occluded the LST data are, since the elevation never changes. The same can be said for the emissivity. Although the emissivity can suffer from occlusion, like the sensors used to derive the LST, the emissivity is fairly constant and can be interpolated linearly over time.

The first step in the statistical reconstruction of LST data is the reconstruction in time. The original work of Metz et al. [6] performs a local weighted regression with polynomial order two, considering the five nearest neighbors in time, with gaps greater than seven days not being interpolated. Extensive hyperparameter tuning via Tree Parzen Estimation [30] and the Optuna framework [31] to optimize on our validation dataset showed that, for our particular use case, polynomials of order five, considering the seven nearest neighbors, and interpolating any gaps up to a size of nine days, improve the results. This could be due to the fact that the weather in our study region is of high variance, compared to the entire region of central Europe, where more moderate climatic conditions are prevalent. Values that cannot be interpolated by the first step are spatially interpolated using thin plate spine (TPS) interpolation [32]. Again, hyperparameter tuning revealed that a relatively strong smoothing coefficient of five helps to achieve the best results, and while the emissivity as a covariable of TPS proved itself helpful for interpolation as suggested in the original work, the elevation was not useful to reduce the interpolation error. This could be explained with the worse results, when the valid information was only accessible in the high- or low-laying parts of our interest region, as elevation as a covariable cannot provide additional information without at least some data points being available for the different value ranges. For a fair comparison, we also added up to 20 value captures from local ground weather stations to the LST images, if they were missing, as they are computed according to Section 2.3.

### 4.2. Deep Learning Parameters

**Figure 4:**
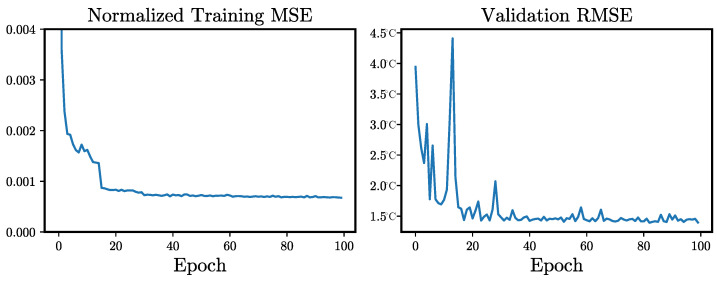
The development of the loss during model training and evaluation. On the left-hand side is the normalized MSE during training with visible improvements after 15 and 30 epochs, when the learning rate is adjusted. On the right-hand side, we see the validation RMSE, calculated on all pixels of the image and therefore being slightly lower than the final evaluation, which is carried out on reconstructed pixels only.

The experiments with our deep interpolation approach are conducted on a NVIDIA Quadro RTX 6000. The batch size during training is set to 6. During training, the well-known Adam optimizer is used [33], with a learning rate of $4\times 10^{-5}$. The learning rate decays by the factor 0.1 after 15 and again after 30 epochs. The network is trained for a total of 100 epochs to guarantee convergence. All parameters are selected on a validation dataset, solely reserved for this purpose, consisting of 140 LST images with no occlusions of the ground truth, and, therefore, all information available. The network input is normalized to the interval [0,1] using the minimum and maximum temperature values of the training dataset. The loss during training and validation can be seen in Figure 4. The training loss improved visibly after 15 and 30 epochs, when the learning rate is adjusted. Both graphs indicate a convergence of the network training without signs of overfitting.

### 4.3. Comparative Evaluation

For the evaluation, two main metrics are used to quantify the performance of the prediction. To measure the overall error, the Root Mean Square Error (RMSE) is calculated as follows:

(2) $$\mathrm{RMSE}\left(\hat{y},y\right)=\sqrt{\mathrm{MSE}(\hat{y},y)}=\sqrt{\frac{1}{n}\sum_{i=1}^{n}{\left({\hat{y}}_{i}-y_{i}\right)}^{2}}.$$

Here, y represents the ground-truth values and $\hat{y}$, the prediction values. To be able to measure the variability in the target variable that is explained by the models, the coefficient of determination (R^2^) is utilized:

(3) $$R^{2}\left(\hat{y},y\right)=1-\frac{\sum_{i=1}^{n}{\left(y_{i}-{\hat{y}}_{i}\right)}^{2}}{\sum_{i=1}^{n}{\left(y_{i}-\bar{y}\right)}^{2}},$$

where $\bar{y}$ is the mean of the ground-truth values *y*. To achieve a fair evaluation, we excluded 140 images with full data coverage as testing data during the entire modeling process. The test images are processed as training samples, explained in Section 3.3, resulting in partially covered images as input to the different approaches but with the full ground truth available. This process of masking pixel values according to the missing value pattern of a randomly selected training example removes an average of about 2500 pixels from the 4012 available pixels per image. The numerical evaluation of those experiments is shown in Table 2, which shows the capabilities of our deep interpolation approach. We improve the RMSE of the statistical approach by 44%, while being able to deliver a gapless reconstruction, even when the only valid pixels available are derived from the inferred data from the local weather stations.

### 4.4. Discussion

**Figure 5:**
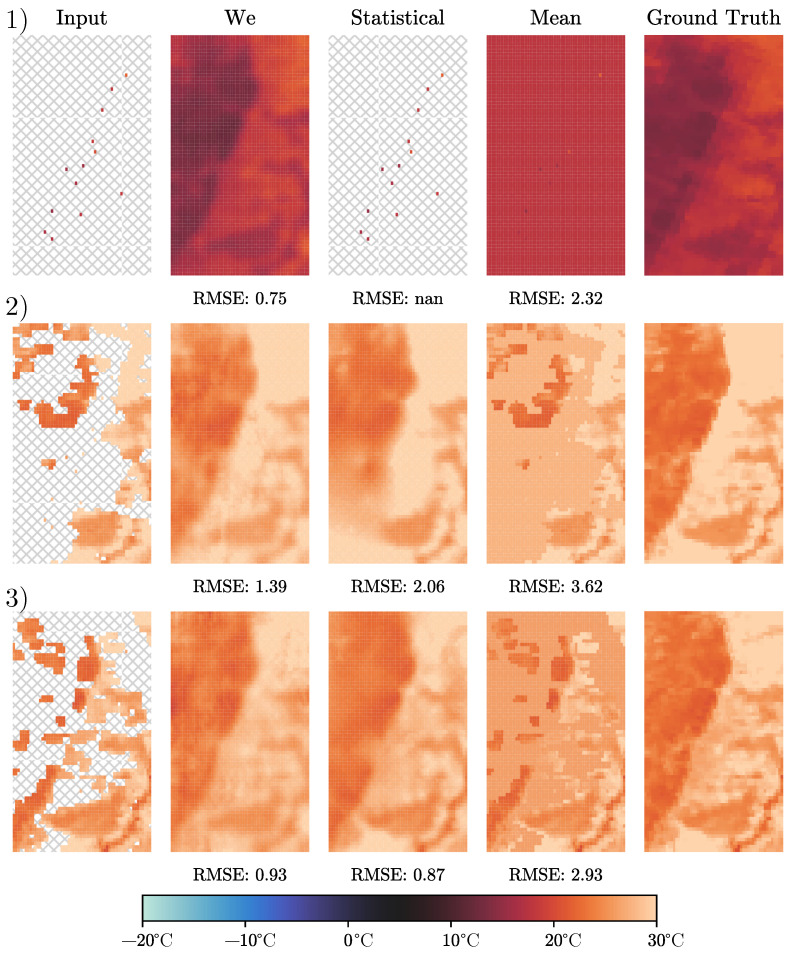
The reconstruction of three typical training images found in our dataset taken on 29 September 2008, 9 May 2008, and 15 April 2008, respectively. In the first row, our research area is fully covered by clouds or cloud shadows, and no LST information is available. In the second row, more data are available, and in the last row, data are available, especially in the challenging area, where the elevation changes drastically, allowing the statistical approach to do a great job of reconstructing the gaps.

When comparing our approach with state-of-the-art statistical methods, we see improvements in two main directions. First, the interpolation always covers 100% of the research area, and second, the general precision is improved from 2.52 °C to 1.67 °C in terms of RMSE. The reason for the first improvement is that the statistical interpolation problem can be ill-posed if only a few data points are available. The first row example in Figure 5 shows a testing instance, where the selected cloud occlusion covers the entire image. Therefore, only pixels with valid information are reconstructed from ground-site weather stations. We see that our deep interpolation approach is capable of reconstructing the whole research area with all its environmental constraints, while the statistical approach is not capable of interpolating. The reason for the second improvement direction can be explained by looking at the second and third examples in Figure 5. The test instance shown in the second row shows a reconstruction error of 2.06 °C for the statistical approach and 1.39 °C for our deep interpolation. When looking at the reconstructed images, we see that the statistical approach is not capable of reconstructing the lower left corner of the image correctly. The ground-truth image shows the steep temperature gradient typical for our research area, which runs from the lower left corner to the upper right corner of the area. The statistical approach cannot reproduce this peculiarity when the information around this gradient is missing as is the case in this example. For the third row example of Figure 5, we observe two very good reconstructions, with error values of 0.93 °C and 0.87 °C for our deep interpolation and the statistical approach, respectively. The high accuracy of the statistical approach can be explained by the existing data in the input image. Although the general number of data points is not too high, the available information is located mostly in the area where the reconstruction is the most challenging, allowing the statistical approach to slightly outperform our deep interpolation. In general, our approach is capable of learning the environmental constraints of the research area and reproducing them, even when crucial information is missing, giving an edge over statistical methods that see each instance as an isolated problem, without being able to induce knowledge from other instances from the research area. One way to help statistical approaches with this problem is to provide additional information in the form of covariables during the interpolation process. The covariables investigated within this study are elevation maps and emissivity. Elevation maps are not included in the results shown in this investigation, as our experiments indicated that including them reduces the accuracy of the reconstruction. However, the emissivity, which is fairly constant and therefore can be easily interpolated, even throughout cloud occlusion, helped to improve the results and added some form of environmental context to the statistical model.

As explained in Section 2.2, the process of obtaining LST information from satellite records is complex. So, naturally, the question arises of whether further processing of the data records with deep learning alters or impacts the data inherently. The first influence on the data comes from the integration of air temperature when interpolating LST. The difference between air temperature and surface temperature varies with respect to different surfaces, which poses a potential source of uncertainty within the interpolation. We minimize the potential for errors by training an individual model for every ground-site weather station and, therefore, allowing the models to account for different surfaces. As shown in Table 1, the results for the different stations are very similar. The question of whether the deep learning pipeline itself alters the data can only be answered by looking at the performance on the test data. The very high correlation between the predicted temperature and real temperature indicated by an R2 score of 0.95 is a clear indication that our approach is true to the data at hand. However, there is a common problem when evaluating LST interpolation, which comes from the fact that the data used for training and evaluating the approaches have the inherent bias of being captured when no clouds are present. This so-called cloud-free assumption is necessary, as it is not possible to achieve ground-truth LST measurements for already occluded data points on a large scale. For our region of interest, the weather station at the ground site also does not provide direct information on LST that could be used to evaluate LST reconstruction without the cloud-free assumption, at least for some selected pixels.

Although the high specialization of our approach to the research area improves accuracy, it is harder to apply when considering a different area. For a new area, the statistical approach can be used out of the box and just needs access to the very instance that needs to be reconstructed. For our deep interpolation, it is necessary to find a database of training examples that can be used to train our deep interpolation before the reconstruction can be applied. However, building such a database is fairly accessible since our proposed learning process can be executed exclusively on occluded ground-truth data as explained in Section 3.3. To fully unlock the potential of deep interpolation in a new research area, the availability of local ground-site weather stations is also necessary. Although the approach would work in most cases without those, we would not be able to provide 100% coverage of the reconstruction. When changing the research area, also the amount of improvement of our deep interpolation approach over the statistical approach might change, as the results shown in Figure 5 indicate the difficult inhomogeneous areas of the region, which is where our approach outperforms the competition.

## 5. Conclusions

Within this research, we used partial convolutions within a U-Net deep learning architecture to interpolate LST data that were retrieved from the observations of the MODIS sensors attached to NASA satellites. Our two-step interpolation approach includes first, the conversion of air temperature data towards LST data, when local ground-site weather stations can provide this information. Second, we use the deep interpolation methods to achieve a gap-free representation of the data. Experiments on our research show that we outperform state-of-the-art statistical approaches by 44% in terms of RMSE. This is achieved through the capabilities of our approach to collect knowledge of the research areas environmental constraints during training, which is missing for the statistical approaches. The results of this work will be used within the federal project KI-iREPro to predict grapevine yields within our study area. Furthermore, we believe that our results prove the capabilities of deep learning approaches for remote sensing LST interpolation and can be adopted to other research areas and use cases around the world.

## Figures and Tables

**Table 1:** Overview of the location of all 20 local ground-site weather stations used to obtain air temperature data. The values of RMSE and R^2^ for the linear regression models that convert the air temperature into LST data.

Name	Latitude	Longitude	RMSE (°C)	R2
Bad Bergzabern	49.11	8.00	2.80	0.92
Deidesheim Niederkirchen	49.43	8.22	2.70	0.94
Edesheim	49.26	8.15	2.82	0.94
Ellerstadt	49.46	8.27	2.47	0.95
Freimersheim	49.27	8.22	2.70	0.92
Goecklingen Holzbruehl	49.16	8.03	2.64	0.93
Herxheimweyher	49.16	8.25	2.88	0.92
Lachen Speyerdorf	49.31	8.20	2.66	0.94
Landau Nussdorf	49.22	8.11	2.75	0.94
Landau Wollmesheim	49.18	8.08	2.60	0.94
Maikammer	49.29	8.15	2.72	0.94
Meckenheim	49.40	8.26	2.77	0.94
Neustadt an der Weinstraße	49.37	8.19	3.07	0.91
Ruppertsberg	49.39	8.19	2.77	0.93
Schaidt	49.05	8.09	2.30	0.93
Schweigen Rechtenbach	49.05	7.97	2.71	0.93
Schweighofen	49.04	7.99	2.48	0.93
Siebeldingen	49.22	8.05	2.67	0.93
Steinweiler	49.10	8.10	2.69	0.93
Wachenheim	49.44	8.19	2.84	0.93

**Table 2:** Results for evaluating the accuracy via RMSE and R2 error metrics on 140 test instances for LST reconstruction. The 140 instances have full coverage of ground-truth data for the day. The value of pixels is masked according to the missing value pattern of a randomly selected training example, removing an average of about 2500 pixels from the 4012 available pixels per image.

Approach	RMSE	R2	Reconstruction
Mean Approximation	3.23 °C	0.81	100%
Statistical Approach [6]	2.52 °C	0.91	52.06%
Deep Interpolation (We)	1.67 °C	0.95	100%

## Data Availability

All data used within this research are publicly available and cited accordingly.
